# Supplementary figures and images for: The emergence of SARS-CoV-2 lineages and associated saliva antibody responses among asymptomatic individuals in a large university community
Source: PLoS Pathog. 2023 Aug 21;19(8):e1011596. doi: 10.1371/journal.ppat.1011596 (PMC10470930; doi:10.1371/journal.ppat.1011596)

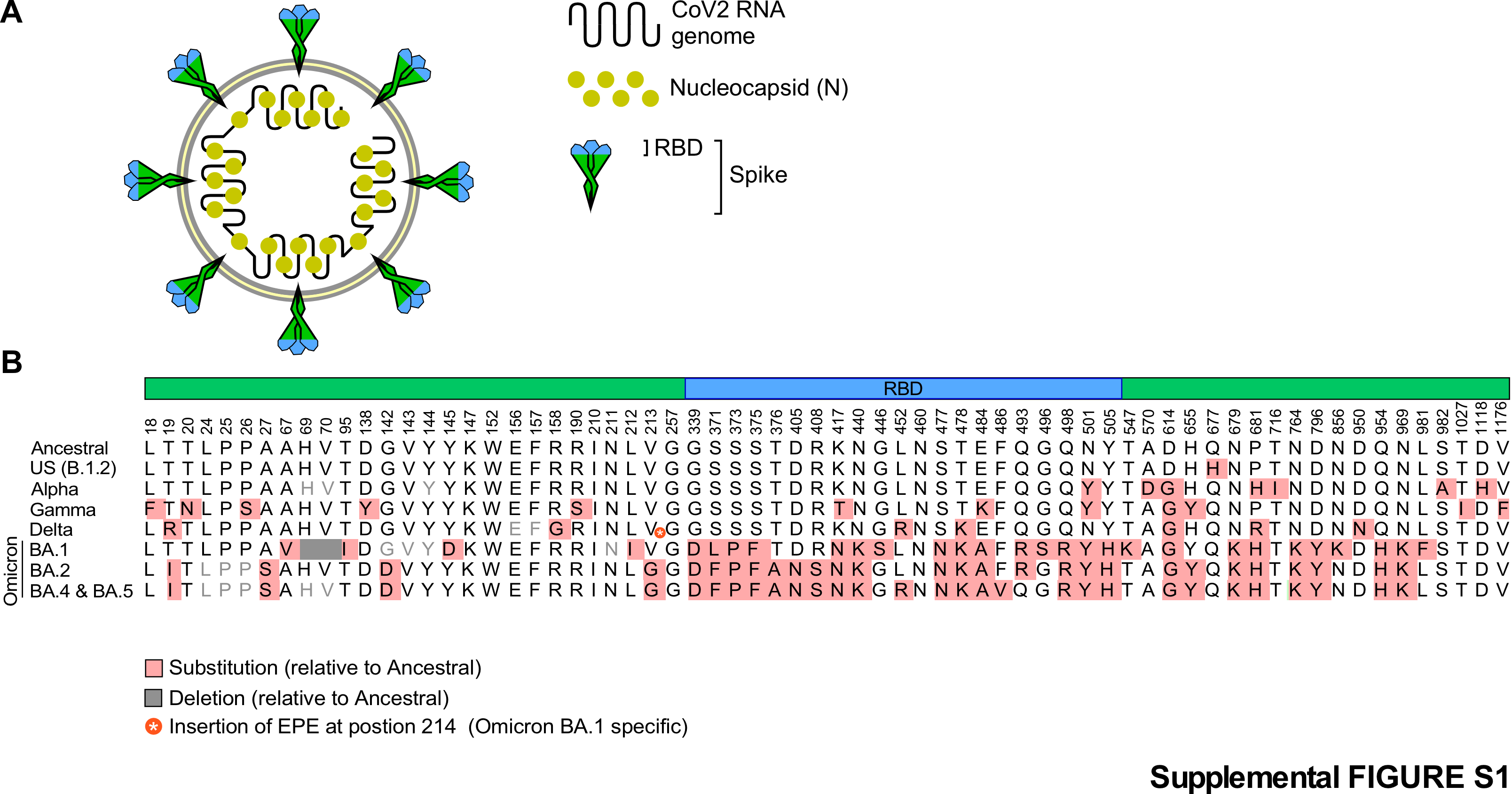

Supplement: S1 Fig — (A) Depiction of CoV2 and its RNA genome, nucleocapsid (N, yellow) and Spike proteins, the latter being differentially colored to indicate the Receptor Binding Domain (RBD, blue) and non-RBD regions (green). (B) The amino acids which distinguish the CoV2Anc Spike protein from CoV2US (also known as B.1.2), CoV2Alpha, CoV2Gamma, and CoV2Delta, as well as the Omicron lineages CoV2O-BA.1, CoV2O-BA.2, CoV2O-BA.4, and CoV2O-BA.5. (TIF) [file ppat.1011596.s001.tif]

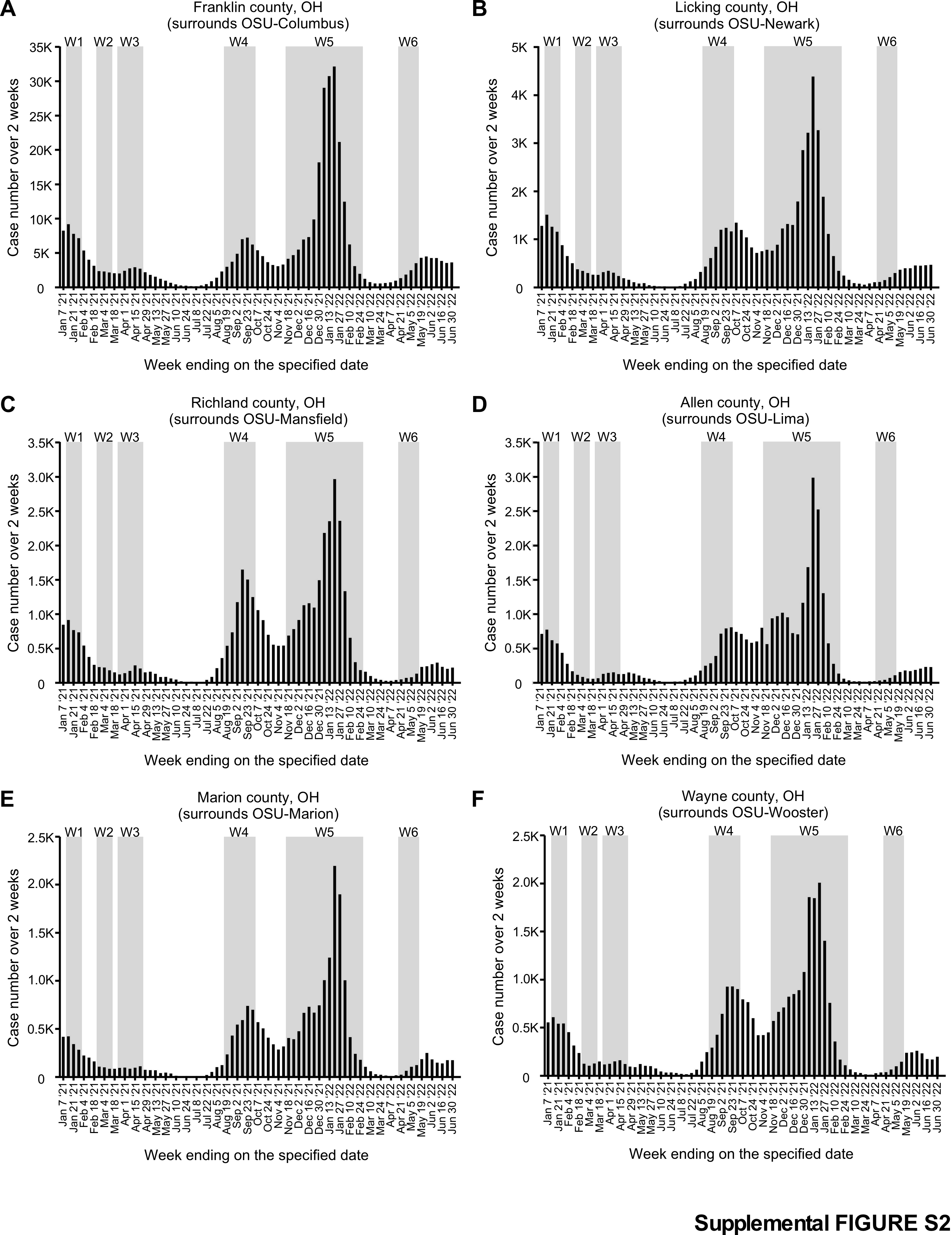

Supplement: S2 Fig — Daily COVID cases in the counties surrounding each campus of our university, as reported by the Ohio Department of Health (ODH), for the period spanning January 2021 to May 2022. Shown are the data for (A) Franklin County, which surrounds the OSU-Columbus campus; (B) Licking County, which surrounds the OSU-Newark campus; (C) Richland County, which surrounds the OSU-Mansfield campus; (D) Allen County, which surrounds the OSU-Lima campus; (E) Marion County, which surrounds the OSU-Marion campus; and (F) Wayne County, which surrounds the OSU-Wooster campus. Overlaid onto each graph are the dates which correspond to the six COVID waves (W1-W6) that occurred in our campus community (see Fig 2). (TIF) [file ppat.1011596.s002.tif]

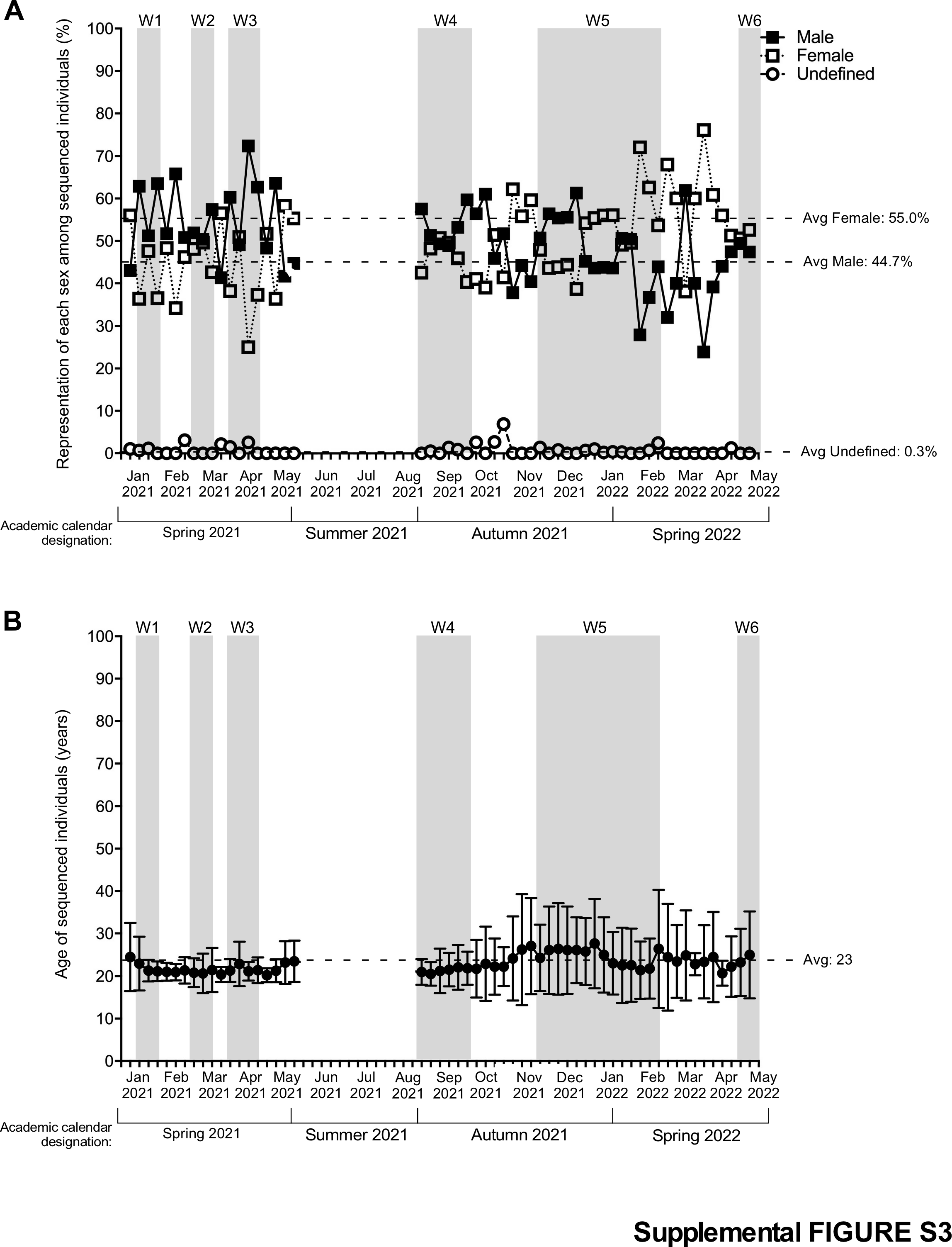

Supplement: S3 Fig — (A) The percent of males, females and undefined sex among individuals whose saliva was PCRPOS and sequenced for lineage identification for each week of our study period, the criteria for sequencing being a CT≤33. The average values for each sex across the entire study period are indicated by the hatched lines. (B) The age range of individuals whose saliva was PCRPOS and sequenced throughout the monitoring period. Overlaid onto each graph in gray are the periods corresponding to Waves 1–6 in our university community along with the academic calendar beginning and end dates. (TIF) [file ppat.1011596.s003.tif]

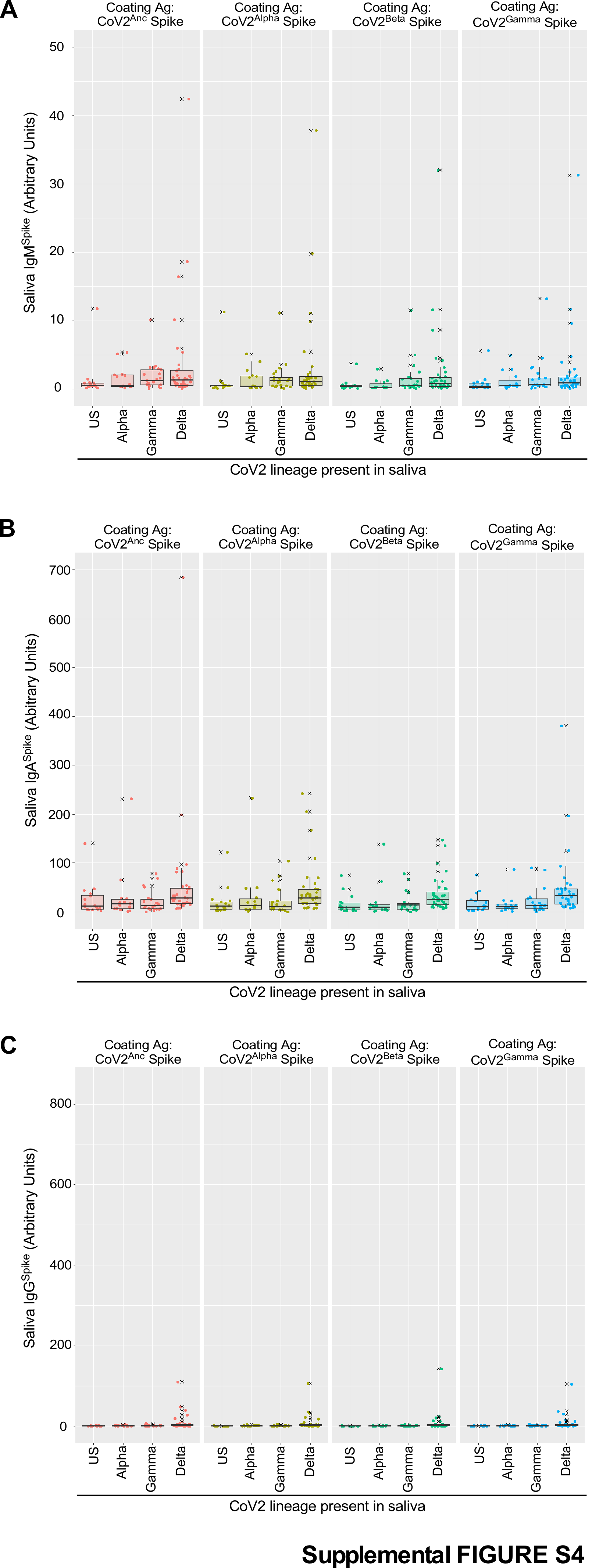

Supplement: S4 Fig — Saliva samples that were positive for either the CoV2US, CoV2Alpha, CoV2Gamma or CoV2Delta lineage were used to measure the concentrations of (A) IgMSpike, (B) IgASpike, and (C) IgGSpike. Varying by column were the coating antigens (Ag) used for each measurement, the Ag being recombinant forms of either the CoVAnc Spike (Column 1), CoV2Alpha Spike (Column 2), CoV2Beta Spike (Column 3), and CoV2Gamma Spike (Column 4). Antibody levels are expressed in arbitrary units of luminescence. Note that the CoVAnc -specific IgM, IgA, and IgG values in (A-C) Column 1 were transformed into WHO Binding Antibody Units (BAUs) for Fig 4. (TIF) [file ppat.1011596.s004.tif]

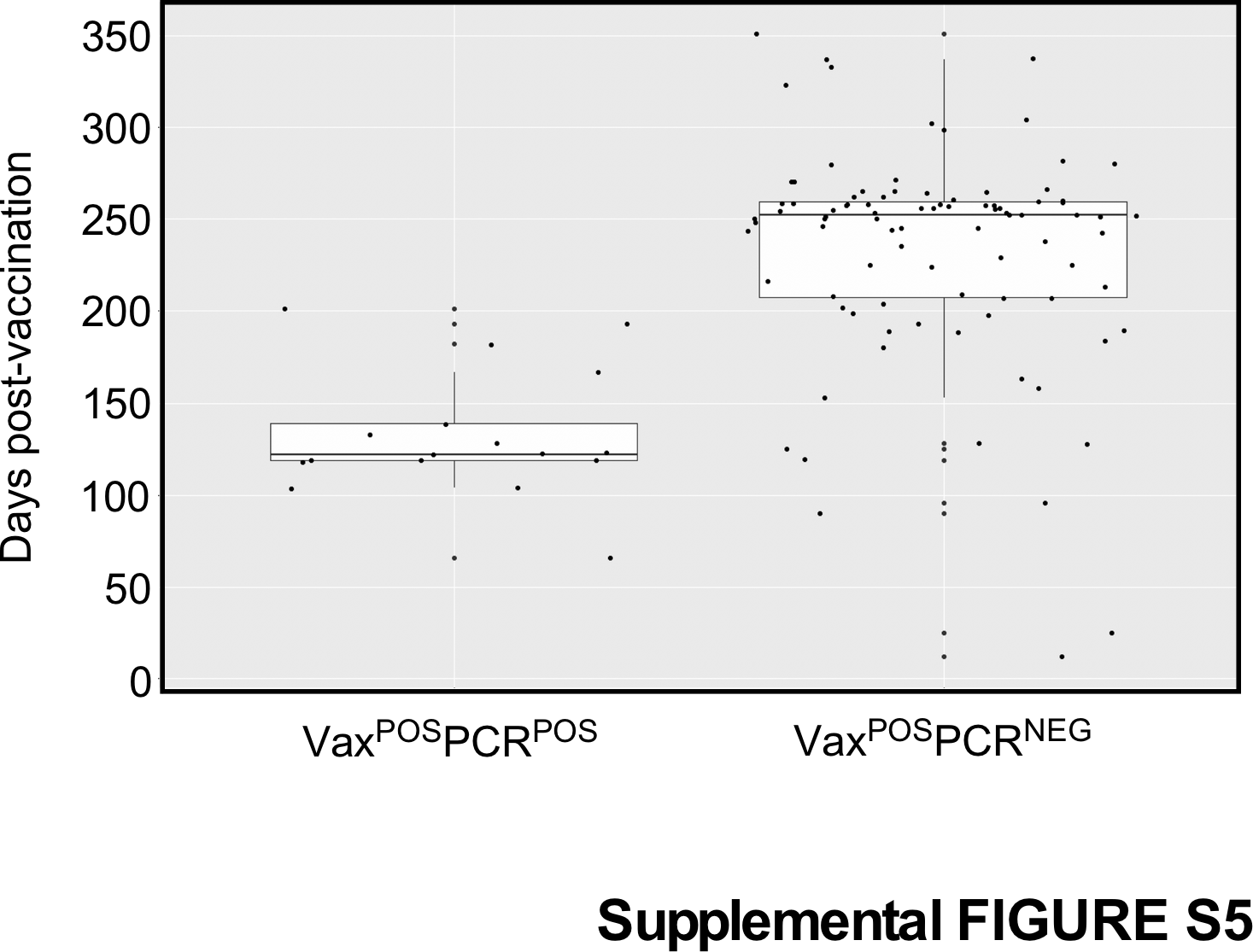

Supplement: S5 Fig — To generate the data shown in Fig 6, we analyzed saliva from individuals with a breakthrough Delta infection (VaxPOSPCRPOS individuals) and those who were vaccinated but PCR negative around the same time (VaxPOSPCRNEG individuals). Shown for both groups are the time in days since receiving the final dose of their vaccine series, with each dot representing a single individual. (TIF) [file ppat.1011596.s005.tif]

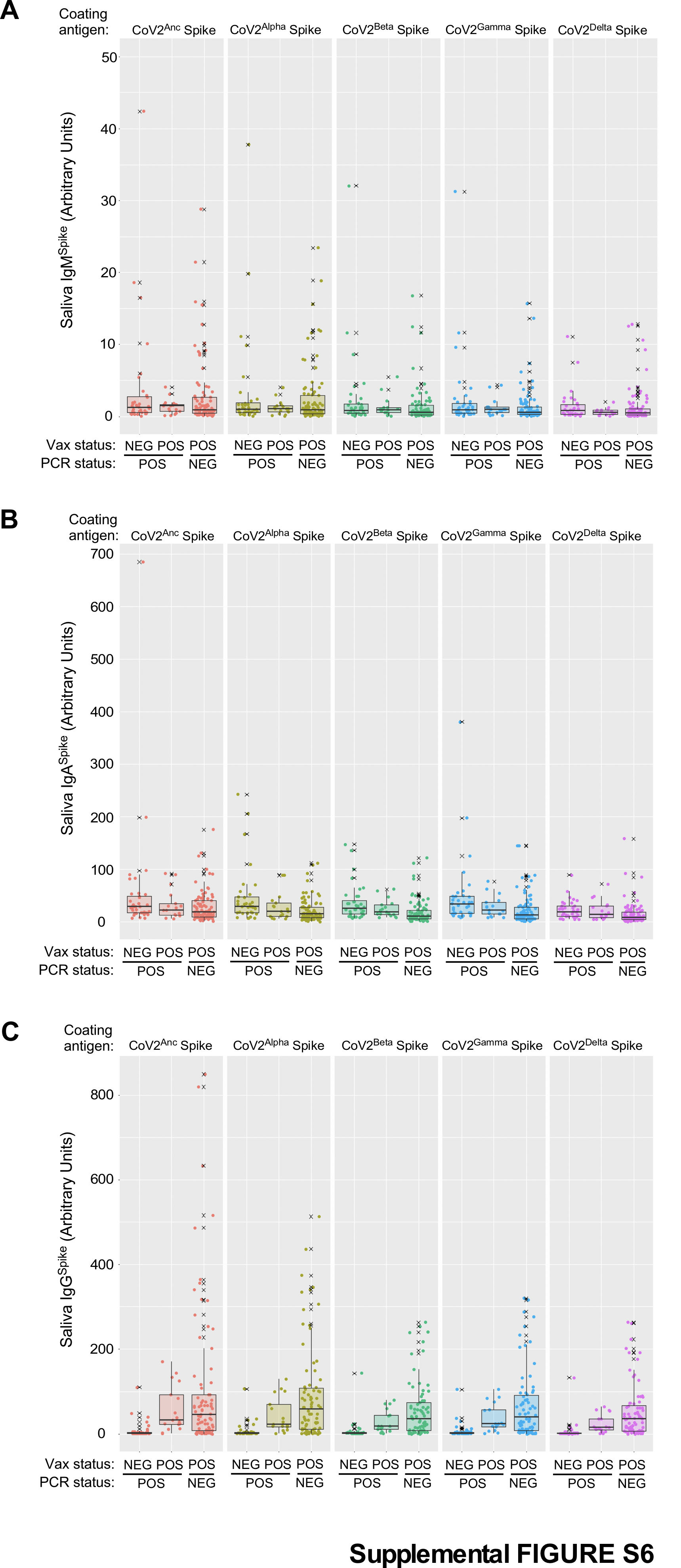

Supplement: S6 Fig — During and after COVID Wave 4 (i.e. that which was caused by CoV2Delta), saliva from three groups of individuals were collected and used for Ig measurements: those who had not been fully vaccinated and were positive for CoV2Delta (VaxNEG PCRPOS), those who had been fully vaccinated and were positive for the CoV2Delta (VaxPOS PCRPOS), and those who had been fully vaccinated and were negative for any CoV2 lineage (VaxPOS PCRNEG). Shown for each individual in each group are the levels of (A) IgMSpike, (B) IgASpike, and (C) IgGSpike which bind to four different coating antigens (Ag), the Ag being recombinant forms of either the CoVAnc Spike (Column 1), CoV2Alpha Spike (Column 2), CoV2Beta Spike (Column 3), CoV2Gamma Spike (Column 4), and CoV2Delta Spike (Column 5). Antibody levels are expressed in arbitrary units of luminescence. Note that for (A) six outliers are not shown, and that the CoVAnc -specific IgM, IgA, and IgG values in (A-C) Column 1 were transformed into WHO Binding Antibody Units (BAUs) for Fig 6. (TIF) [file ppat.1011596.s006.tif]
